# The repair of the Achilles tendon rupture: comparison of two percutaneous techniques

**DOI:** 10.1007/s11751-011-0124-1

**Published:** 2011-11-08

**Authors:** G. Taglialavoro, C. Biz, G. Mastrangelo, R. Aldegheri

**Affiliations:** 1 Orthopaedic and Traumatologic Clinic, Department of Medical and Surgical Specialties, Padua University, via Giustiniani 3, 35128 Padua, Italy; 2 Epidemiology Laboratory, Department of Occupational Medicine, Padua University, via Giustiniani 3, 35128 Padua, Italy

**Keywords:** Achilles tendon, Rupture, Percutaneous technique, Tenolig, Ma and Griffith

## Abstract

This study proposes a comparison between two percutaneous techniques of subcutaneous Achilles tendon rupture by evaluating the risk of lesion developing, the morbidity of the surgical technique adopted and the effectiveness of each technique. Sixty patients were operated at Padua Orthopaedic Clinic by using the two different procedures: (1) Ma and Griffith in 30 cases and (2) Tenolig in 30 cases. Risk of rupture developing has been evaluated in relation to sex, age, side, kind of trauma, work and presence of preoperative risk factors. The Morbidity of surgical technique has been evaluated in with respect to surgical time, hospital permanence, immobilization, active nonweight-bearing mobilization, assisted weight bearing until the full one, number of early and late complications before and after hospital discharge. Effectiveness has been evaluated in relation to return time to common life, work and sport; anatomical and functional features have been evaluated using McComis score, rating results as: very good (from 80 to 70), good (from 69 to 60), fair (from 59 to 50) and poor (<50). Tenolig group shows shorter average time from hospital admission and operation, hospital permanence and immobilization (*P* < 0.05), and it results in an easier and quicker execution and functionally stimulates the tendon healing in a short time. Effectiveness was the same for both techniques because average McComis score was good (*P* = 0.35), and there was no significant differences in common life returning time (*P* = 0.12). Tenolig technique seems to be preferable to Ma and Griffith.

## Introduction

Subcutaneous Achilles tendon rupture is a meta-traumatic injury that occurs in a clinical context in which a trauma, generally ineffective to cause the lesion, acts on an altered tendon already degenerated because of the constitutional robustness of the subject, related to contributory factors such as age, gender, lifestyle and sport activities [1].

The incidence is increasing [2], particularly in amateur athletes (“weekend warriors”) [3], who are involved in acceleration/deceleration activities and do not warm-up sufficiently for training in comparison with professional athletes.

The most affected age is between 30 and 40 with a prevalence of males (9:1) [4].

This injury has been the subject of many studies, but its causes and treatments are still controversial. Some authors prefer functional treatments [5, 6], although this seems to provide a higher re-rupture incidence [7]. However, nowadays, the greater number of experts prefers the mini-invasive surgical treatment to open surgery, because the first is associated with a smaller number of complications and it is demonstrated to be more respectful to the biology of the tendon cicatrization [8, 9].

Currently at our institution, the treatment of choice for this kind of injury is based on two different percutaneous techniques: the Ma and Griffith (M&G) suture and the Tenolig (T) one.

The purpose of the present study is to:

- Assess the *risk* of lesions developing;
- Analyze the *morbidity* of the surgical technique adopted;
- Demonstrate and compare the *effectiveness* of each technique.

## Methods

We performed a retrospective cohort study on 60 consecutive patients who were surgically treated for acute Achilles tendon rupture from 01/01/2005 to 26/05/2010. The choice of surgical technique was determined by the personal preference of the surgeon.

The clinical data were obtained from the medical records of each patient, during the period from 1/3/2010 to 31/6/2010.

The final clinical control was performed from 1/8/2010 to 31/8/2010, calling the patients for a follow-up at our institution.

The population was divided into 2 groups based on the two different percutaneous techniques adopted:

- Ma and Griffith (M&G) suture [10] in 30 cases.
- Tenolig (T) suture [11] in 30 cases.

### Surgical technique

*The Ma and Griffith percutaneous suture technique* requires the execution of between 6 and 8 small incisions at the sides of the proximal and distal stump of the tendon, in order to be able to direct and cross the wire suture.

**Fig. 1:**
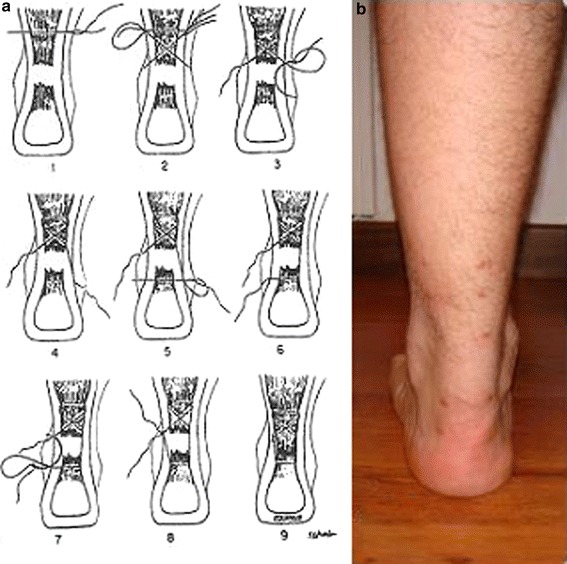
**a** Ma and Griffth technique schematic description; **b** Follow-up at 90 days from surgery

This technique does not shorten the time of immobilization and it is not usually performed in Day-Surgery. In fact, the wire used does not allow the patient an early mobilization. A below-knee plaster cast is applied with the ankle plantar-flexed for 30 days and then a second cast with the ankle in neutral position for 20 days. Returning to sports activities is permitted after 12 weeks (Fig. 1).

*The Tenolig device* consists of a thread with a diameter of 0.85 mm and a length of 36 cm, onto which a 7-m-wide metal harpoon is mounted, crimped at its distal end by a 15-cm-long flexible triangular-tipped needle and an anchoring system.

Two small skin incisions are performed on the sides of the proximal portion of the tendon, approximately 6 cm above the rupture zone. The sural aponeurosis is displayed.

The needle is then inserted, taking care to penetrate into the proximal and distal portion of the tendon and then is pushed out at the sides of the calcaneal tuberosity, 4 or 5 cm below the rupture.

**Fig. 2:**
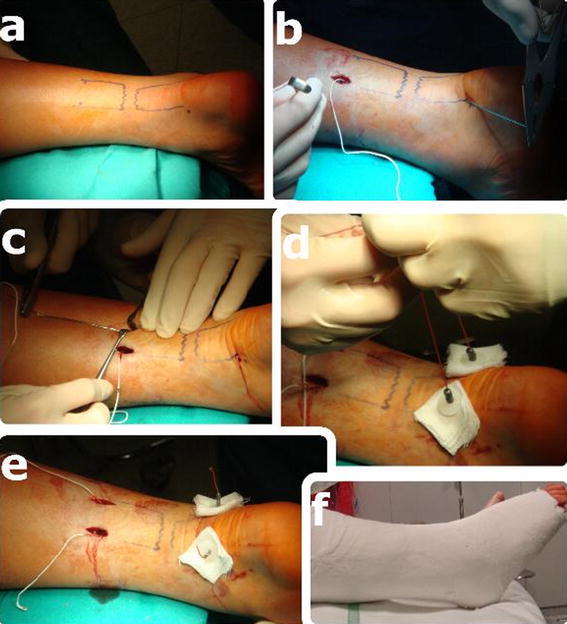
**a** The position of the ruptured tendon is marked; **b** The first needle is inserted; **c** The second one is inserted taking care to penetrate the tendon perpendicularly; **d** The two straps are pulled tight simultaneously; **e** The plastic buttons and the weights are threaded in order to fix the device; **f** A below-knee plaster cast is applied

The metal wire harpoon penetration (2 sharpened branches) is directed and orientated so that it is then connected to the proximal portion of the sural aponeurosis. The suture wires are tensed with the foot in the equine position, and the anchoring system is applied (Fig. 2).

The postoperative treatment provides for a below-knee plaster cast with the ankle plantar-flexed from the 1st to the 21st day, an articulated walking boot from the 22nd to the 45th day and removal of the Tenolig device on the 45th day. Resumption of full unsupported weight bearing is gradually permitted from the 45th to the 90th day, and return to sports activities is possible after 90 days.

*Risk* of lesion developing was assessed in relation to sex, age, side, kind of trauma, kind of job and presence of preoperative risk factors.

*Morbidity* of surgical treatment relative to each technique was evaluated considering the time necessary for surgery, duration of hospitalization, immobilization, active nonweight-bearing mobilization, assisted weight bearing until full weight bearing without any help and the number of early and late complications before and after hospital discharge.

*Effectiveness* was analyzed by observing the time required for return to normal daily life, work and sports; the McComis score [12] was used for the anatomical and functional analysis (Table 1). Accordingly, results are rated as: very good (scoring from 80 to 70), good (69 to 60), fair (59 to 50) and poor (<50).

**Table 1:** The McComis score used for the anatomical and functional analysis

McComis anatomical and functional analysis	Score
*Dorsiflexion (compared with opposite side)*	
Not increased	10
Decreased of 5°	5
Decreased or equal to 10°	1
Decreased more than 10°	0
*Plantar flexion (compared with opposite side)*	
Not increased	10
Decreased of 5°	5
Decreased or equal to 10°	1
Decreased more than 10°	0
*Circumference of sural muscle 10* *cm below the knee (compared with opposite side)*	
No differences	10
Difference at <1 cm	8
Difference at <2 cm	6
Difference at <3 cm	4
*Laid plantar (compared with opposite side measuring the distance between heel and ground)*	
Normal	10
Decreased of 50%	5
Sketchy	1
Impossible	0
*Pain*	
Absent	10
During intense training	8
During moderate training	4
Continuous	0
*Recovery of sports activity*	
Complete	10
Low loss	8
Decreased	6
Not recovered	4
Complaints during normal activity	0
*Satisfaction of the patient*	
Very good	10
Good	8
Fair	6
Poor	0

For statistical analysis, two patients who were operated on using the Tenolig technique were excluded because they did not participate in the follow-up study.

For univariate analysis, we encoded the two surgical techniques by a single dichotomous variable that means 1 for M&G and 0 for T; to assess the risk of injury developing, some indicators were recoded in dichotomous variables:

- Sex notations are: 1 (males) and 0 (females);
- Side of the lesion: 1 (right) and 0 (left);
- Efficient traumatic context (playing football, basketball, volleyball, tennis, pushing a broken car, falling down the stairs or from a stool, slipping on the street, in the garden, in a hotel) 1;
- Inefficient traumatic context (when it occurred during mild domestic housekeeping or it was apparently spontaneous, walking, dancing) 0;
- Profession notation: 1 when it did not involve any manual and heavy work (white collar such as lawyer, clerk, shop assistant, draftsman, professional, dealer, retired person, salesman, student, housewife/husband, engineer, dental technician, hotel doorkeeper), and 0 when it involved heavy work (blue collar such as worker or metalworker but also prisoner, sportsman);
- Presence of previous diseases: 1 (diabetes, tendonitis, diseases that required the use of quinolone, patients who previously underwent tendon corticosteroids infiltration or previously reported Achilles tendon contralateral breakage, smokers); 0 if the patient was a healthy person.

Active nonweight-bearing mobilization was not included in the analysis of surgical treatment morbidity, because it was done only by patients treated with the Tenolig technique. Complications before and after hospital discharge: 1 if present and 0 if absent. To verify the effectiveness and compare between the two techniques, the time (days) it took to return to work and sports was not included because some retired patients and other patients who never did sport were present. The McComis score was recoded in a polytomous variable (results of functionality) rated as: 3 for very good functionality, 2 for good, 1 for fair and 0 for poor.

Given the limited number of patients, we used nonparametric tests: the Wilcoxon test (numeric variables) and the χ^2^ test (categorical variables) for one-way analysis of variance.

Subsequently, multiple linear regression analysis (stepwise forward) was performed to select the variables affecting the treatment effectiveness when the dependent variable was a numeric one (obtaining the regression coefficient, the confidence interval up to 95% and the level of significance), and multiple logistic regression analysis (exact method) was performed when the dependent variable was a categorical one (getting the odds ratio, the confidence interval up to 95% and the level of significance). In the linear regression model, independent variables were only those of risk of injury developing and surgical treatment morbidity. In the logistic regression model, independent variables were those associated with lesion risk, expressed as dichotomous variables. Indeed, the presence of an empty cell in the table “trauma versus complications” needed the use of the multiple logistic regression “exact method.” The large amount of memory required by this algorithm calculation has limited the number of variables that could enter into the model.

## Results

The average follow-up after operation was 610.75 (±183.32) days in the M&G group, while it was 770.67 (±301.96) days in the T group (Table 2). The difference between methods is at the limit of statistical significance (*P* = 0.06).

**Table 2:** Risk of lesion developing analysis

	Ma and Griffith		Tenolig		*P* value
	Media (DS)	Num. (%)	Media (DS)	Num. (%)	
Follow-up (day)	610.75 (182.32)		770.67 (301.96)		0.06
Age (year)	40.40 (11.54)		44.85 (18.71)		0.32
Sex (male = 1)		25 (85%)		25 (91%)	0.34
Side (right = 1)		14 (45%)		13 (45%)	0.97
Trauma contest (efficient = 1)		25 (85%)		25 (91%)	0.34
Profession (white collar = 1)		25 (85%)		21 (76%)	0.57
Preoperative risk (yes = 1)		14 (45%)		7 (25%)	0.14

The average patient age in the M&G group was 40.40 (±11.54) and 44.85 (±18.71) in T, again with negligible statistical differences (*P* = 0.32).

In both groups, males were more frequently affected: 85% of the cases (25 patients) in the M&G group and 91% (25 cases) in the T group, without any statistically significant difference (*P* = 0.34).

The right side was affected in 45% of the cases in both groups (M&G 14 cases, T 13 cases) again without any statistically significant difference (*P* = 0.97).

Traumatic context was mainly efficient in 85% of the cases in the M&G group (25 cases) and 91% (25 cases) in the T group, without any statistically significant difference (*P* = 0.34).

Lifestyle was mainly sedentary, as in most cases patients were white collar workers: 85% of the cases (25 cases) in the M&G group and 76% (21 cases) in the T group, even if no statistically significant difference (*P* = 0.57) was observed.

Risk factors were present in 45% (14 cases) of the patients treated according to M&G technique and in 25% (7 cases) of patients treated according to T technique, with no statistical difference between the groups (*P* = 0.14). Risk factors were smoking (10 cases), previous tendonitis (9 cases), previously reported Achilles tendon contralateral breakage (3 cases), diabetes (2 case) and local corticosteroid infiltrations (1 case).

The average hospitalization was 4.25 days (±1.51) in the M&G group and 2.94 days (±2.94) in the T group (Table 3). This difference is significant (*P* = 0.01), although it may be due to a longer time between hospital admission and operation in the M&G group (average 2.2 ± 1.6 days), while in the T group, it was 1.19 (±0.92) days, again with a significant difference (*P* = 0.03).

**Table 3:** Technique morbidity analysis

	Ma and Griffith		Tenolig		*P* value
	Media (DS)	Num. (%)	Media (DS)	Num. (%)	
Operation waiting time (days)	2.2 (1.6)		1.19 (0.92)		0.03
Operation time (min)	29.4 (11.89)		30.31 (10.13)		0.38
Hospital permanence (days)	4.25 (1.51)		2.94 (1.39)		0.01
Immobilization (days)	46.5 (9.10)		28.28 (8.62)		0.00
Assisted weight bearing time (days)	30.15 (0.48)		28.53 (6.44)		0.17
Late complications (yes = 1)		4 (15%)		9 (33%)	0.18

The average surgical procedures execution time was about 30 min (*P* = 0.38) in both cases.

All patients received a spinal anesthesia.

The average immobilization time was significantly higher (*P* = 0.001) in the M&G group with the patients wearing a cast for 46.5 (±9.10) days in contrast to the T patients who were immobilized for an average time of 28.28 (±8.62) days.

The assisted weight bearing was 30 days on average for both techniques (*P* = 0.17); after that full weight bearing was granted. There have never been early complications.

In patients operated on with the M&G technique, late complications occurred in 15% (4 cases) of the cases always as transitional sensitive sural neuropathy. On the other hand, in patients operated with T technique, complications occurred in 33% (9 cases) of the cases, including 2 review of the percutaneous suture after spontaneous re-rupture, 2 sensitive transient sural neuropathy, 2 shoe conflict, 1 hook retention at the time of removal, 1 partial retention of suture wire and 1 infection following the removal. However, this late complication difference was not statistically significant (*P* = 0.18).

Return to ordinary life occupations was on average 100.30 (±29.25) days in the M&G group and 88.23 ± (48.90) days in the T group with no statistically significant differences (*P* = 0.12) (Table 4).

**Table 4:** Technique effectiveness analysis

	Ma and Griffith	Tenolig	*P* value
	Media (DS)	Media (DS)	
Common life return time (days)	100.30 (29.25)	86.23 (48.90)	0.12
McComis score	66.30 (7.18)	65.12 (16.16)	0.35
Functional results (score)	2.25 (0.62)	2.19 (0.90)	0.62

The McComis score was on average 66.30 (±7.18) in the M&G group and 65.12 (±16.16) in the T group; this difference was not statistically significant (*P* = 0.35).

In both groups, the clinical functionality is virtually equivalent (M&G 2.25 ± 0.62 and T 2.19 ± 0.90), and this difference was not statistically significant (*P* = 0.62).

With multivariate analysis, we observed that the return to normal life occupations was influenced by profession (*P* = 0.01) as white collar patients returned to common life occupations 41 days earlier than blue collar patients (Table 5).

**Table 5:** Multiple linear regression analysis (stepwise forward)

Model variable	Coeff.	PR 95%	*P* value
Profess (white collar = 1)	−41.15	−71.67, −10.62	0.01
Technique (Ma and Griffith = 1)	23.01	−1.58, 47.60	0.06
Costant	80.38	37.50, 123.24	0.00

Dependent variable: common life return time (days after operation). Regression coefficient (Coeff), power ranges 95% (PR 95%), *P* value for a two tail test

In the same multiple linear regression model, the McComis score and the clinical functionality are not influenced by any of the considered independent variables (data not shown); hence, both techniques provide the same result in terms of treatment effectiveness.

In the logistic multiple regression model, the number of late complications is significantly influenced (*P* = 0.02) by the preoperative risk factors (Table 6).

**Table 6:** Multiple logistic regression analysis (exact method)

Model variable	OR	PR 95%	*P* value
Technique (Ma and Griffith = 1)	0.17	0.00–1.97	0.23
Trauma contest (efficient = 1)*	4.80	0.42–+Inf	0.21
Preoperative risk (yes = 1)	13.6	1.22–775.74	0.02
Operation waiting time (days)	0.96	0.45–1.87	1.00
Sex (male = 1)	1.31	0.07–93.75	1.00

Dependent variable: Late complications. regression coefficient (Coeff), power ranges 95% (PR 95%), *P* value for a two tail test

* *MUE* median unbiased estimates

## Discussion

On the basis of the results reported previously, the following considerations can be proposed:

1. With respect to the *risk* of developing a subcutaneous rupture of the Achilles tendon, we observed a higher predisposition to Achilles tendon rupture in those patients who practice noncompetitive sports [1], who are male, middle-aged, involved in a sedentary lifestyle, smokers, or have undergone corticosteroid local infiltrations [13], or had previous local tendonitis and with previous contralateral Achilles tendon injury [14]. These results are in agreement with what has already been reported in the literature.
2. The technique *morbidity* in the two groups shows no statistically significant differences as far as the number of developed complications is concerned. The coexistence of preoperative risk factors, like smoking, tendonitis, local corticosteroid infiltrations, metabolic diseases like diabetes, increase the chance of the occurrence of late complications. This could be explained because they lead to the disruption [15] of tendon fibers, peripheral neurolability and micro-vascular failure. However, in two cases, there is a correlation between technique and late complications: a hook retention case and a partial retention of suture wire case occurred in the Tenolig removal operation which caused superficial infection; in both cases, the problem was solved with medication and antibiotic therapy.

However, the low number and the kind of complications recorded, the functional result obtained (for both techniques they are generally good) and the return to normal life demonstrate that the techniques compared in this study are minimally aggressive and very efficient.

3. In relation to the *effectiveness*, a comparison between the techniques indicates equal anatomical and functional outcomes for both as they are generally good and there was no significant difference in the return time to ordinary life. However, white collar workers returned earlier to work than others because our patients were professionals and self-employed workers who needed to recover faster in order to support their families, while blue collar workers were less sensitive to this problem.

## Conclusion

In conclusion, we believe that the surgical treatment, and in particular the percutaneous suture, represents the gold standard for the treatment of acute ruptures of the Achilles tendon as it is minimally aggressive, respectful to the biology of the tendon cicatrization and devoid of serious complications. In fact, the percutaneous suture guarantees a strong primary stabilization of the stumps of the ruptured tendon (just one case of re-rupture on 60 operated patients).

However, the authors of the present study prefer the Tenolig technique because functionally it stimulates the tendon healing in a short time, already after the third week, favouring the tendon cicatrization processes. The Tenolig technique also results in an easier and quicker execution with a lower risk of damage to the sural nerve, compared to the Ma and Griffith technique.

In fact, the latter technique requires the execution of between six and eight small incisions at the sides of the proximal and distal stump of the tendon, in order to be able to direct and cross the wire suture that contains a high risk of sural nerve injury. The wire used does not allow the patient an early mobilization. This technique does not shorten the time of immobilization, and it is not usually performed in Day-Surgery.

Conversely, the Tenolig suture requires the execution of two small skin incisions on the sides of the proximal portion of the tendon.

The needle on which the wire is mounted is inserted along the longitudinal axis of the tendon and rarely affects the sural nerve. Using the metallic anchor, the traction exerted on the proximal portion of the tendon is made along the longitudinal axis of the tendon, so the hauling effect on the proximal stump approaching the distal stump reproduces similar physiological results with respect to the forces that normally ensure the proper function of the tendon and its subsequent healing. The very strong wire allows the early mobilization of the tendon from the third postoperative week and partial supported weight bearing is also permitted early. Finally, the Tenolig technique is usually performed in Day-Surgery with an incremental relationship between costs and benefits.
